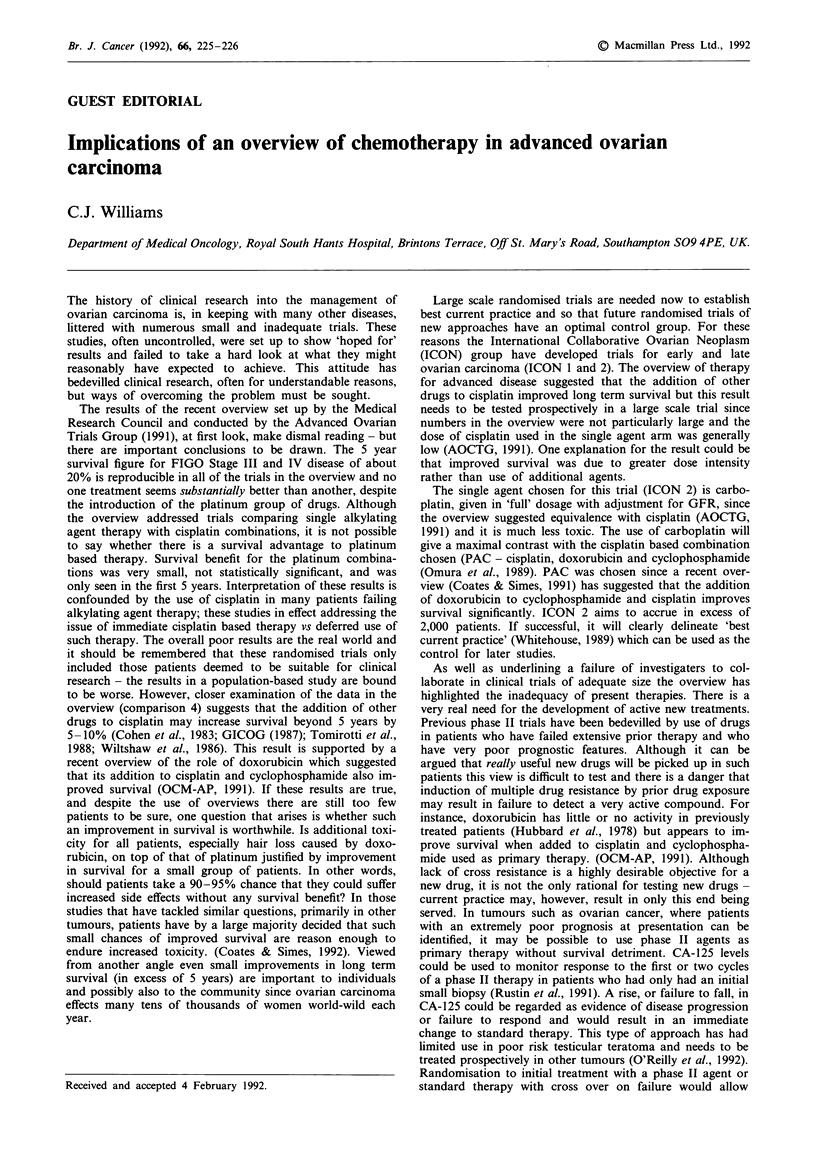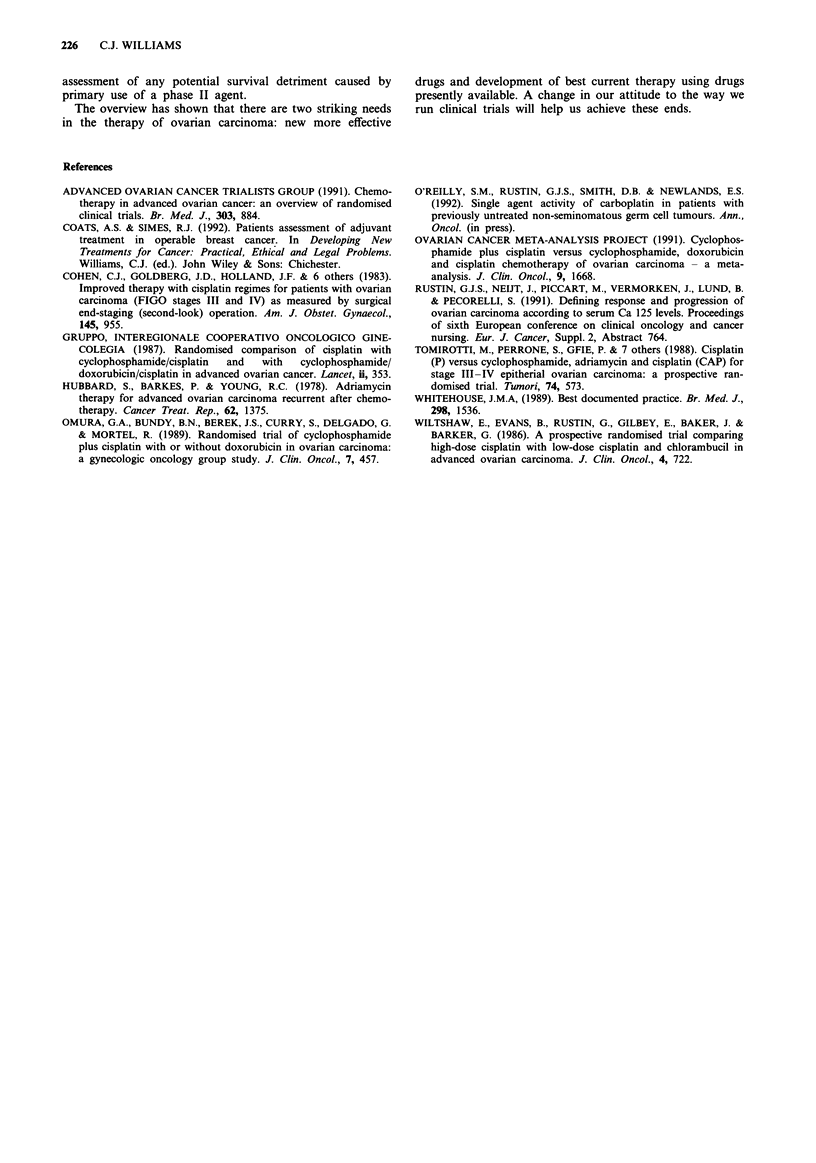# Implications of an overview of chemotherapy in advanced ovarian carcinoma.

**DOI:** 10.1038/bjc.1992.248

**Published:** 1992-08

**Authors:** C. J. Williams


Br. J. Cancer (1992), 66, 225–226 © Macmillan Press Ltd., 1992

GUEST EDITORIAL

### Implications of an overview of chemotherapy in advanced ovarian carcinoma

C.J. Williams

*Department of Medical Oncology, Royal South Hants Hospital, Brintons Terrace, Off St. Mary's Road, Southampton SO9 4PE, UK.*

The history of clinical research into the management of ovarian carcinoma is, in keeping with many other diseases, littered with numerous small and inadequate trials. These studies, often uncontrolled, were set up to show 'hoped for' results and failed to take a hard look at what they might reasonably have expected to achieve. This attitude has bedevilled clinical research, often for understandable reasons, but ways of overcoming the problem must be sought.

The results of the recent overview set up by the Medical Research Council and conducted by the Advanced Ovarian Trials Group (1991), at first look, make dismal reading – but there are important conclusions to be drawn. The 5 year survival figure for FIGO Stage III and IV disease of about 20% is reproducible in all of the trials in the overview and no one treatment seems *substantially* better than another, despite the introduction of the platinum group of drugs. Although the overview addressed trials comparing single alkylating agent therapy with cisplatin combinations, it is not possible to say whether there is a survival advantage to platinum based therapy. Survival benefit for the platinum combinations was very small, not statistically significant, and was only seen in the first 5 years. Interpretation of these results is confounded by the use of cisplatin in many patients failing alkylating agent therapy; these studies in effect addressing the issue of immediate cisplatin based therapy *vs* deferred use of such therapy. The overall poor results are the real world and it should be remembered that these randomised trials only included those patients deemed to be suitable for clinical research – the results in a population-based study are bound to be worse. However, closer examination of the data in the overview (comparison 4) suggests that the addition of other drugs to cisplatin may increase survival beyond 5 years by 5–10% (Cohen *et al.*, 1983; GICOG (1987); Tomirotti *et al.*, 1988; Wiltshaw *et al.*, 1986). This result is supported by a recent overview of the role of doxorubicin which suggested that its addition to cisplatin and cyclophosphamide also improved survival (OCM-AP, 1991). If these results are true, and despite the use of overviews there are still too few patients to be sure, one question that arises is whether such an improvement in survival is worthwhile. Is additional toxicity for all patients, especially hair loss caused by doxorubicin, on top of that of platinum justified by improvement in survival for a small group of patients. In other words, should patients take a 90–95% chance that they could suffer increased side effects without any survival benefit? In those studies that have tackled similar questions, primarily in other tumours, patients have by a large majority decided that such small chances of improved survival are reason enough to endure increased toxicity. (Coates & Simes, 1992). Viewed from another angle even small improvements in long term survival (in excess of 5 years) are important to individuals and possibly also to the community since ovarian carcinoma effects many tens of thousands of women world-wild each year.

Received and accepted 4 February 1992.

Large scale randomised trials are needed now to establish best current practice and so that future randomised trials of new approaches have an optimal control group. For these reasons the International Collaborative Ovarian Neoplasm (ICON) group have developed trials for early and late ovarian carcinoma (ICON 1 and 2). The overview of therapy for advanced disease suggested that the addition of other drugs to cisplatin improved long term survival but this result needs to be tested prospectively in a large scale trial since numbers in the overview were not particularly large and the dose of cisplatin used in the single agent arm was generally low (AOCTG, 1991). One explanation for the result could be that improved survival was due to greater dose intensity rather than use of additional agents.

The single agent chosen for this trial (ICON 2) is carboplatin, given in 'full' dosage with adjustment for GFR, since the overview suggested equivalence with cisplatin (AOCTG, 1991) and it is much less toxic. The use of carboplatin will give a maximal contrast with the cisplatin based combination chosen (PAC – cisplatin, doxorubicin and cyclophosphamide (Omura *et al.*, 1989). PAC was chosen since a recent overview (Coates & Simes, 1991) has suggested that the addition of doxorubicin to cyclophosphamide and cisplatin improves survival significantly. ICON 2 aims to accrue in excess of 2,000 patients. If successful, it will clearly delineate 'best current practice' (Whitehouse, 1989) which can be used as the control for later studies.

As well as underlining a failure of investigaters to collaborate in clinical trials of adequate size the overview has highlighted the inadequacy of present therapies. There is a very real need for the development of active new treatments. Previous phase II trials have been bedevilled by use of drugs in patients who have failed extensive prior therapy and who have very poor prognostic features. Although it can be argued that *really* useful new drugs will be picked up in such patients this view is difficult to test and there is a danger that induction of multiple drug resistance by prior drug exposure may result in failure to detect a very active compound. For instance, doxorubicin has little or no activity in previously treated patients (Hubbard *et al.*, 1978) but appears to improve survival when added to cisplatin and cyclophosphamide used as primary therapy. (OCM-AP, 1991). Although lack of cross resistance is a highly desirable objective for a new drug, it is not the only rational for testing new drugs – current practice may, however, result in only this end being served. In tumours such as ovarian cancer, where patients with an extremely poor prognosis at presentation can be identified, it may be possible to use phase II agents as primary therapy without survival detriment. CA-125 levels could be used to monitor response to the first or two cycles of a phase II therapy in patients who had only had an initial small biopsy (Rustin *et al.*, 1991). A rise, or failure to fall, in CA-125 could be regarded as evidence of disease progression or failure to respond and would result in an immediate change to standard therapy. This type of approach has had limited use in poor risk testicular teratoma and needs to be treated prospectively in other tumours (O'Reilly *et al.*, 1992). Randomisation to initial treatment with a phase II agent or standard therapy with cross over on failure would allow assessment of any potential survival detriment caused by primary use of a phase II agent.

The overview has shown that there are two striking needs in the therapy of ovarian carcinoma: new more effective drugs and development of best current therapy using drugs presently available. A change in our attitude to the way we run clinical trials will help us achieve these ends.

### References

ADVANCED OVARIAN CANCER TRIALISTS GROUP (1991). Chemotherapy in advanced ovarian cancer: an overview of randomised clinical trials. *Br. Med. J.*, **303**, 884.

COATS, A.S. & SIMES, R.J. (1992). Patients assessment of adjuvant treatment in operable breast cancer. In *Developing New Treatments for Cancer: Practical, Ethical and Legal Problems.* Williams, C.J. (ed.). John Wiley & Sons: Chichester.

COHEN, C.J., GOLDBERG, J.D., HOLLAND, J.F. & 6 others (1983). Improved therapy with cisplatin regimes for patients with ovarian carcinoma (FIGO stages III and IV) as measured by surgical end-staging (second-look) operation. *Am. J. Obstet. Gynaecol.*, **145**, 955.

GRUPPO, INTEREGIONALE COOPERATIVO ONCOLOGICO GINECOLEGIA (1987). Randomised comparison of cisplatin with cyclophosphamide/cisplatin and with cyclophosphamide/doxorubicin/cisplatin in advanced ovarian cancer. *Lancet*, **ii**, 353.

HUBBARD, S., BARKES, P. & YOUNG, R.C. (1978). Adriamycin therapy for advanced ovarian carcinoma recurrent after chemotherapy. *Cancer Treat. Rep.*, **62**, 1375.

OMURA, G.A., BUNDY, B.N., BEREK, J.S., CURRY, S., DELGADO, G. & MORTEL, R. (1989). Randomised trial of cyclophosphamide plus cisplatin with or without doxorubicin in ovarian carcinoma: a gynecologic oncology group study. *J. Clin. Oncol.*, **7**, 457.

O'REILLY, S.M., RUSTIN, G.J.S., SMITH, D.B. & NEWLANDS, E.S. (1992). Single agent activity of carboplatin in patients with previously untreated non-seminomatous germ cell tumours. *Ann., Oncol.* (in press).

OVARIAN CANCER META-ANALYSIS PROJECT (1991). Cyclophosphamide plus cisplatin versus cyclophosphamide, doxorubicin and cisplatin chemotherapy of ovarian carcinoma – a meta-analysis. *J. Clin. Oncol.*, **9**, 1668.

RUSTIN, G.J.S., NEIJT, J., PICCART, M., VERMORKEN, J., LUND, B. & PECORELLI, S. (1991). Defining response and progression of ovarian carcinoma according to serum Ca 125 levels. Proceedings of sixth European conference on clinical oncology and cancer nursing. *Eur. J. Cancer*, Suppl. 2, Abstract 764.

TOMIROTTI, M., PERRONE, S., GFIE, P. & 7 others (1988). Cisplatin (P) versus cyclophosphamide, adriamycin and cisplatin (CAP) for stage III–IV epitherial ovarian carcinoma: a prospective randomised trial. *Tumori*, **74**, 573.

WHITEHOUSE, J.M.A, (1989). Best documented practice. *Br. Med. J.*, **298**, 1536.

WILTSHAW, E., EVANS, B., RUSTIN, G., GILBEY, E., BAKER, J. & BARKER, G. (1986). A prospective randomised trial comparing high-dose cisplatin with low-dose cisplatin and chlorambucil in advanced ovarian carcinoma. *J. Clin. Oncol.*, **4**, 722.